# Application of synthetic computed tomography based on a deep learning model in pelvic radiotherapy planning: A feasibility study

**DOI:** 10.1002/acm2.70829

**Published:** 2026-09-28

**Authors:** Feng Lin, Jinyan Hu, Zhao Li, Jun Wang, Wenying Zhang, Aiping Wen, Mao Li, Yunfei Hu, Yang Wang

**Affiliations:** ^1^ Radiotherapy Oncology Center Jinshazhou Hospital of Guangzhou University of Chinese Medicine Guangzhou China; ^2^ Chengdu Branch Siemens Healthineers Digital Technology (Shanghai) Co., Ltd. Chengdu China; ^3^ Icon Cancer Centre Gosford Gosford Australia; ^4^ University of Wollongong Wollongong Australia

**Keywords:** deep learning, MRI‐only workflow, pelvic radiotherapy, synthetic CT

## Abstract

**Background:**

While computed tomography (CT) remains an important imaging modality in radiotherapy planning, magnetic resonance imaging (MRI)‐only workflow, often realized via the generation of synthetic computed tomography (sCT), is desired due to superior soft‐tissue contrast and less susceptibility to registration errors.

**Purpose:**

The aim of this study was to investigate the feasibility of applying sCT, generated from MRI using a deep learning model, to pelvic radiotherapy planning. By comparing differences between conventional CT and sCT in terms of image quality, dosimetric parameters, and three‐dimensional (3D) dose distributions, the clinical potential of an MRI‐only radiotherapy workflow was evaluated.

**Methods:**

Fifteen patients who underwent both pelvic CT and MRI examinations were retrospectively enrolled. For the same patients, sCT images were generated from the MRI using a pretrained pelvic deep learning model integrated in the syngo.via VB60A software platform. Treatment plans originally created on planning CT (pCT) images were re‐calculated on corresponding sCT datasets. Subsequently, the mean absolute error (MAE) of CT numbers, Dice similarity coefficients (DSCs) for major structures, dose‐volume histogram (DVH) parameters for the planning target volume (PTV) and major organs at risk (OARs), and 3D dose distributions were compared between plans calculated on pCT and sCT.

**Results:**

In terms of image quality, sCT demonstrated comparable CT number accuracy with pCT for soft tissue structures, with MAEs ranging from 6.8 to 8.5 Hounsfield units (HU) for the PTV, bladder, and rectum. Higher MAEs were observed for bony structures (femoral heads and bone marrow), with average MAEs ranging between 17 and 19 HU. The DSC was 0.94 ± 0.01 for the right femoral head, 0.93 ± 0.01 for the left femoral head, and 0.85 ± 0.03 for bone marrow. Regarding dosimetric comparison, the mean absolute differences in key dosimetric parameters for the PTV (D98%, D95%, and Dmean) were all below 0.6%, remaining within clinically acceptable limits. Among the OARs, except for relatively large differences for the maximum and minimum doses in the bladder, the mean dose (Dmean) differences for the rectum, femoral heads, and bone marrow were minimal (mean absolute value ≤ 0.34%), indicating high reliability. Analysis of 3D dose distributions using a 2% 2 mm γ criterion revealed an average γ passing rate of 95.92% between sCT and pCT, satisfying routine clinical quality assurance requirements.

**Conclusions:**

The findings of our study demonstrate the feasibility of applying sCT generated from MRI using a deep learning model to pelvic radiotherapy planning. The MRI‐only workflow proposed on this basis eliminates systematic errors introduced by CT‐MRI image registration, optimizes the clinical workflow, and fully exploits the superior soft‐tissue contrast of MRI to improve target delineation accuracy. The proposed approach provides a novel technical paradigm for achieving more precise and efficient individualized radiotherapy.

## 1 INTRODUCTION

Among different imaging modalities, computed tomography (CT) remains the foundation of radiotherapy planning. CT images not only provide superior geometric fidelity and high spatial resolution but also supply electron or mass density information required for dose calculation.^1^, ^2^, ^3^, ^4^, ^5^, ^6^, ^7^ The major limitation of CT imaging is its insufficient soft‐tissue contrast. In anatomical regions such as the brain, head and neck, and pelvis, grayscale differences between tissues on CT images are often minimal and tissue boundaries are indistinct, thereby increasing uncertainties in target volume and organ‐at‐risk (OAR) delineation. This directly affects treatment efficacy and increases the risk of radiation‐induced toxicities in OARs.^8^, ^9^, ^10^, ^11^ Magnetic resonance imaging (MRI), on the other hand, provides superior soft‐tissue contrast.^12^, ^13^ However, incorporating MRI into radiotherapy planning introduces additional systematic errors, particularly from the registration between MRI and CT images, where uncertainties can reach up to 5 mm.^14^, ^15^, ^16^, ^17^, ^18^ One way to mitigate this error is to generate synthetic CT (sCT) from MRI images, thereby enabling dose calculation without the involvement of CT images.

Signal intensity in MRI reflects proton density and magnetic relaxation characteristics and does not directly associate with the electron or mass density of tissue. Therefore, the generation of sCT is essentially a complex mapping process. Existing approaches proposed in the literature to estimate electron density maps from MR images include bulk segmentation, atlas‐based methods, and machine learning‐based techniques employing algorithms such as generative adversarial networks (GANs) and convolutional neural networks (CNNs).^9^, ^19^ Among these techniques, increasing attention has been directed to the use of deep learning algorithms to generate sCTs due to their demonstrated efficiency and accuracy.^20^, ^21^, ^22^ Additionally, commercial solutions based on deep learning models have emerged, the performance of which has been evaluated and compared in literature. For example, in a study by Autret et al.,^23^ four commercial sCT generators, namely Syngo_BD, MRI Planner, Syngo_AI, and Therapanacea sCT, were compared within large cohorts of brain and pelvic patients. Their evaluation of image quality demonstrated that deep learning methods achieved superior Hounsfield unit (HU) accuracy compared with the bulk segmentation method.

Notably, the performance of deep learning models in converting MRI images into sCT depends heavily on the quantity and quality of training datasets.^24^, ^25^ Additionally, they are subject to uncertainties arising from data noise and limited generalizability to previously unseen data. These uncertainties may translate into errors in the resultant sCT images and subsequently affect calculation accuracy. In this study, sCT images generated from the MRI using a commercial deep learning model integrated in the syngo.via VB60A software platform were comprehensively assessed in terms of image quality, HU accuracy, geometric fidelity, dosimetric parameters, and three‐dimensional (3D) dose distributions. The aim was to evaluate the feasibility of applying an MRI‐only workflow based on this deep learning model to pelvic radiotherapy planning.

## 2 MATERIALS AND METHODS

### 2.1 Clinical data

A total of 15 patients who underwent both CT and MRI examinations for the pelvis at the Jinshazhou Hospital of Guangzhou University of Chinese Medicine in 2015 were randomly selected. The interval between the two examinations was kept within 18 hours to minimize anatomical variations in OARs such as bladder and rectum. All patient data was anonymized to ensure privacy.

### 2.2 Image acquisition

CT scans were performed using a Siemens SOMATOM Confidence large‐bore CT scanner (Siemens Healthineers, Forchheim, Germany). The scans were acquired with a tube voltage of 120 kVp, a tube current of 400 mA, a field of view (FOV) of 500 mm, and a slice thickness of 2 mm. MRI scans were performed on a Siemens MAGNETOM Vida 3.0T MRI scanner (Siemens Healthineers, Forchheim, Germany) using a T1‐weighted volumetric interpolated breath‐hold examination (VIBE) Dixon sequence. The main MRI parameters included a repetition time (TR) of 4.12 ms, an echo time 1 (TE1) of 1.23 ms, a flip angle of 10°, a FOV Head of 500 mm, a FOV Phase of 75%, and a slice thickness 2 mm. A body coil dedicated to radiotherapy planning was used during the scan. The acquisition time for the pelvic MRI was approximately 3 min. All patients were positioned on a flat couch in the head‐first supine position and were immobilized using a vacbag or a thermoplastic mask during both CT and MRI simulations.

Subsequently, in‐phase and opposed‐phase MRI images were generated. This technique exploits the difference in precessional frequencies between hydrogen protons in water and fat molecules, causing the two signals to periodically exhibit in‐phase and opposed‐phase states. Through mathematical reconstruction, four image sets can be generated, i.e., in‐phase (water + fat), opposed‐phase (water − fat), water‐only (in‐phase + opposed‐phase), and fat‐only (in‐phase − opposed‐phase) images. Osseous structures were reconstructed using a multi‐atlas model, while soft tissues were classified into water or fat categories based on spectral information. Air regions were segmented using a threshold‐based method.^26^, ^27^, ^28^

### 2.3 sCT generation

Following MRI acquisition, sCT images were generated using the deep learning algorithm integrated within the syngo.via VB60A software platform. This algorithm is not a diffusion model but rather a two‐stage deep learning pipeline. Specifically, the model uses the in‐phase and opposed‐phase T1‐weighted VIBE MRI images as the input. A densely connected U‐net first segments the input images into three tissue classes, namely background, bone, and soft tissue. A conditional generative adversarial network (cGAN) subsequently uses both the MRI images and the segmentation output from the previous step to generate sCT images with continuous HU values. An iterative training process was performed by the vendor using 6486 paired brain CT‐MR datasets and 9059 paired pelvic CT‐MR datasets to ensure the accuracy of the model.^26^ Generation of the sCT using this deep learning model takes approximately 5 min. Notably, the training and validation of this model was completed by the vendor and does not require any user input. In the present study, the HU to relative electron density (RED) conversion table (Table 1) provided by the vendor was applied to dose calculations on the generated sCT dataset.

**TABLE 1:** HU to RED conversion table.^26^

Tissue Type	HU value	RED
Air	−1000.000	0.000
Fat	−100.000	0.924
Water	0.000	1.000
Brain/Muscle	40.000	1.040
Spongy bone	200.000	1.096
Cortical bone	1150.000	1.695

### 2.4 sCT assessment

A rigid registration, guided by bony structures, was first performed between the patient's planning CT (pCT) and sCT. Following registration, the PTV, originally delineated by the radiation oncologist (RO), was propagated from the pCT to the sCT using the same rigid registration matrix. Meanwhile, OARs on the pCT and sCT, including the bladder, rectum, bone marrow, and femoral heads, were delineated using the AccuContour 4.0.7.2 software (MANTEIA, Xiamen, Fujian, China). These contours were subsequently reviewed and revised by the same RO before being approved for planning use‌.

Next, dosimetric assessment was conducted using the patient's original treatment plan created on the pCT. All treatment plans were generated using a dual‐arc volumetric modulated arc therapy (VMAT) technique with a 6 MV flattening filter‐free (FFF) beam on the TrueBeam linear accelerator platform (Varian Medical Systems, Palo Alto, CA, USA). Prescriptions for the PTV ranged from 1.8 Gy to 3.0 Gy per fraction, delivered over 15 to 25 fractions, with total doses ranging from 45 Gy to 50 Gy. Plans were created using the AXB16120 algorithm in the Eclipse 16.1 treatment planning system (Varian Medical Systems, Palo Alto, CA, USA) and calculated using a grid size of 0.25 cm. Treatment plans were subsequently propagated to the sCT using the same rigid registration matrix and recalculated using the same algorithm and grid.

To assess the performance of the sCT, evaluations were carried out in terms of HU accuracy, geometric fidelity, and dosimetric accuracy, assessed in both dose‐volume histogram (DVH) metrics and 3D γ‐analysis.

CT HU accuracy was assessed using the mean absolute error (MAE) of HU values for the PTV and OARs between the pCT and sCT. The MAE was calculated using the following formula^29^, ^30^, ^31^:

$$MAE=\left|\bar{HU_{sCT}}-\bar{HU_{CT}}\right|,$$

where $\bar{HU_{sCT}}$ refers to the mean HU value of a structure on the sCT and $\bar{HU_{CT}}$ represents the mean HU value of the corresponding structure on the pCT.

Geometric fidelity was evaluated using the Dice similarity coefficient (DSC) of bony structures between the pCT and sCT. Notably, soft‐tissue OARs, namely bladder and rectum, were excluded from the DSC analysis. This is because despite the short interval between the acquisitions of pCT and MRI, the volume of these two structures may nonetheless differ significantly due to differences in intestinal gas distribution and bladder filling. The DSC is calculated using the following formula, with values closer to 1 indicating higher overlap between two structures.^32^

$$DCS=\frac{2\left|V_{CT}\cap V_{sCT}\right|}{\left|V_{CT}\right|+\left|V_{sCT}\right|}\;,$$

where $V_{CT}$ denotes the volume of the structure on the pCT, $V_{sCT}$ denotes the volume of the corresponding structure on the sCT, and $V_{CT}\cap V_{sCT}$ represents the overlapping volume of the same structure between the two datasets.

The calculation accuracy of the sCT was assessed using both DVH metrics and 3D γ‐analysis. Differences in DVH metrics were calculated for both the PTV and OARs using the following formula.

$$\;Dose_{diff}=\frac{DVH_{sCT}-DVH_{CT}}{prescription\;dose}\;,$$

where $DVH_{sCT}$ represents the DVH metric obtained from the plan created on the sCT, $DVH_{CT}$ refers to the corresponding metric from the plan calculated on the pCT, and “*prescription dose*” denotes the prescribed dose for the plan.

Additionally, a 3D γ‐analysis was conducted between the dose distributions on the pCT and sCT using criteria of 2% 2 mm, 2% 1 mm, 1% 2 mm, and 1% 1 mm. The comparison was performed using absolute doses, with a 5% threshold and global normalization.

### 2.5 Statistical analysis

All statistical analyses were performed using SPSS version 22. A paired‐sample t‐test was used to evaluate the differences between the two groups when the data approximately followed a normal distribution; otherwise, the Wilcoxon signed‐rank test was applied. A p‐value < 0.05 was considered statistically significant. The results were presented using boxplots and described as mean ± standard deviation (x ± s).

## 3 RESULTS

The pCT, sCT, and MRI images of a representative patient are shown in Figure 1. In general, anatomical details on the sCT are highly comparable to those on the pCT. Nonetheless, small differences are observed between the two datasets, especially in the bone and rectum. Additionally, minor blurring on the edge of tissue and bony structures is noted on the sCT.

**FIGURE 1:**
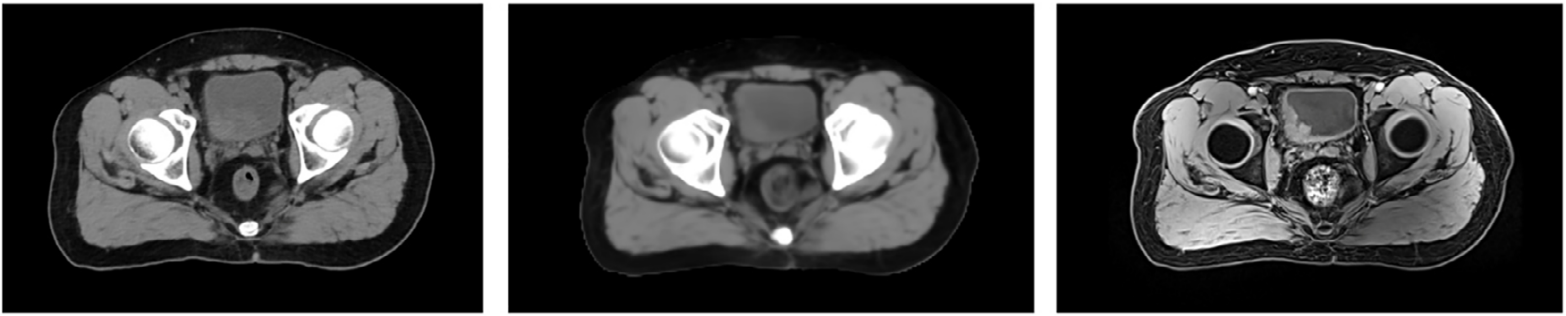
pCT, sCT, and MRI images of a representative patient. Left: pCT; Middle: sCT; Right: T1 VIBE MRI.

The dose distributions of a representative plan on the pCT and sCT are shown on Figure 2.

**FIGURE 2:**
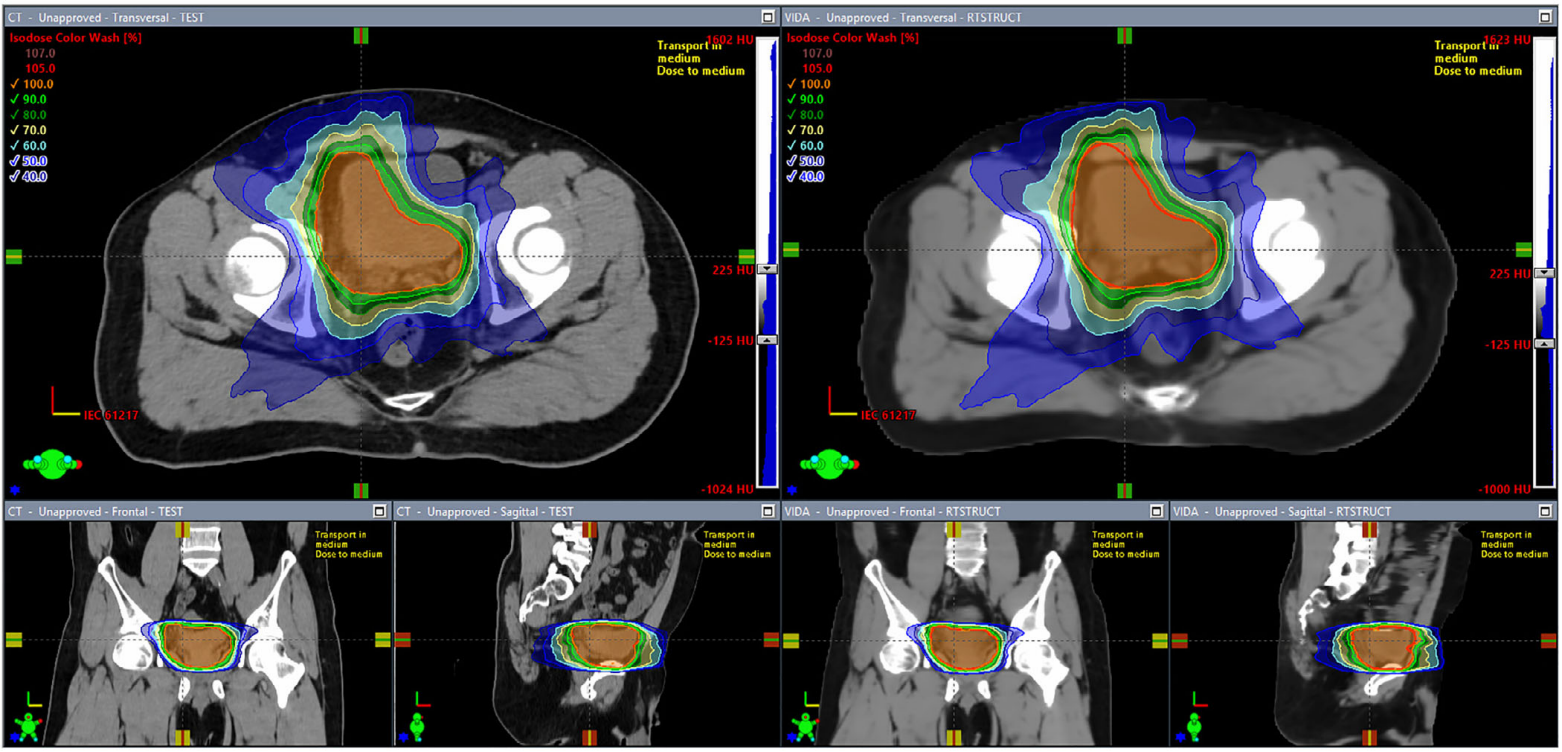
Dose distributions on the pCT and sCT. Left: pCT; Right: sCT.

### 3.1 HU accuracy

Figure 3 presents the boxplots of the CT HU MAE distributions for different structures between pCT and sCT. The MAE for the PTV ranged from 0.149 to 21.166 HU, with a mean value of 6.786 ± 7.883 HU (*P* = 0.239). The rectal MAE ranged from 1.566 to 18.033 HU, with a mean value of 8.126 ± 6.048 HU (*P* = 0.241). The bladder MAE ranged from 0.002 to 23.083 HU, with a mean value of 8.452 ± 7.929 HU (*P* = 0.959). The MAE for the right femoral head ranged from 0.346 to 51.170 HU, with a mean value of 18.702 ± 19.698 HU (*P* = 0.285), while that for the left femoral head ranged from 0.753 to 56.738 HU, with a mean value of 17.420 ± 19.934 HU (*P* = 0.285). Bone marrow MAE ranged from 1.215 to 43.788 HU, with a mean value of 17.440 ± 14.847 HU (*P* = 0.646). No statistically significant differences in mean CT numbers were observed between pCT and sCT for any evaluated structure.

**FIGURE 3:**
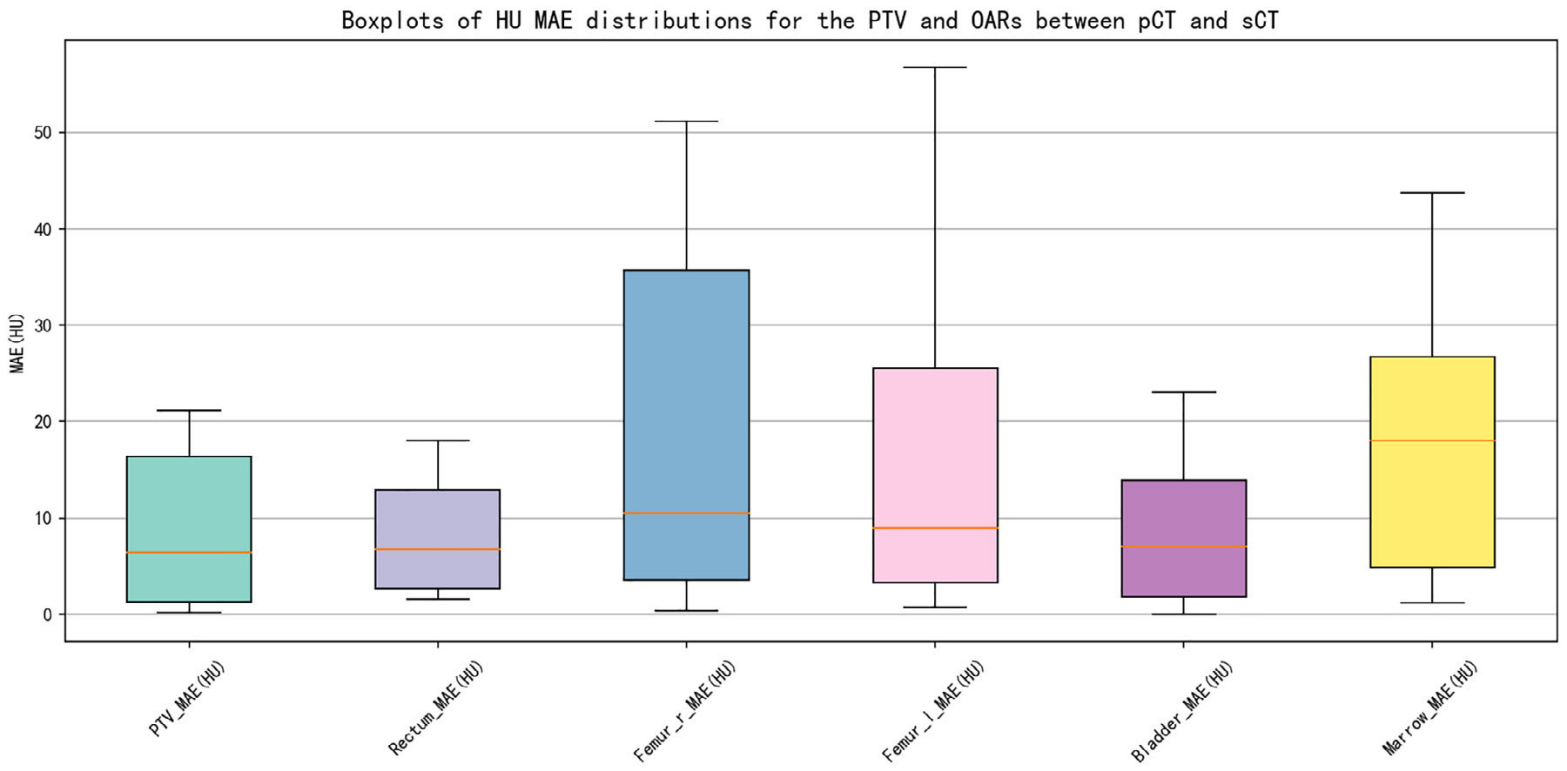
Boxplots of HU MAE distributions for the PTV and OARs between pCT and sCT.

### 3.2 Geometric fidelity

The DSCs for bony OARs, i.e., right femoral head, left femoral head, and bone marrow, are listed in Table 2. All DSCs are above 0.8, indicating high degree of agreement between contours delineated on pCT and those generated on sCT.^33^

**TABLE 2:** DSCs for right femoral head, left femoral head, and bone marrow.

Structure	R Femoral Head	L Femoral Head	Bone Marrow
DSC	0.94 ± 0.01	0.93 ± 0.01	0.85 ± 0.03

### 3.3 Comparison of DVH Metrics for the PTV

Differences in the DVH metrics for the PTV between the pCT and sCT are depicted in Figure 4. Specifically, the mean difference was ‐0.51% ± 1.18 (*P* = 0.144) for PTV Dmin, ‐0.09% ± 0.83 (*P* = 0.508) for PTV D98%, 1.12% ± 1.22 (*P* = 0.075) for PTV Dmax, 0.79% ± 1.04 (*P* = 0.074) for PTV D2%, 0.57% ± 1.01 (*P* = 0.139) for PTV Dmean, and 0.34% ± 0.99 (*P* = 0.646) for PTV D95%. No statistically significant differences were observed in the DVH metrics for the PTV between plans calculated on the pCT and those on the sCT.

**FIGURE 4:**
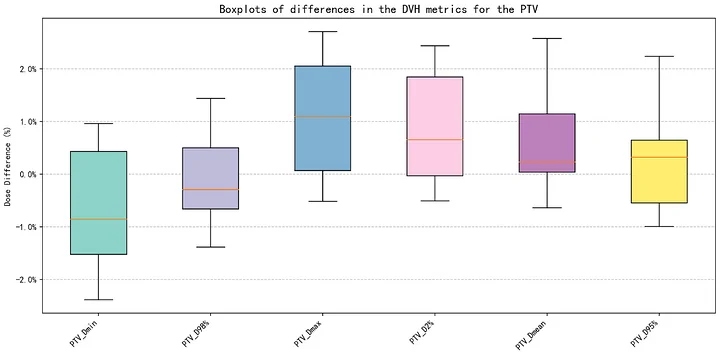
Boxplots of differences in the DVH metrics for the PTV between the pCT and sCT.

### 3.4 Comparison of DVH metrics for OARs

Figure 5 and 6 show differences in the DVH metrics for various OARs. Specifically, for the rectum, the mean differences were ‐0.24% ± 1.51 for Dmin (*P* = 0.646), 0.32% ± 1.45 for Dmax (*P* = 0.575), and 0.34% ± 0.85 for Dmean (*P* = 0.386). For the bladder, the mean differences were ‐1.53% ± 3.77 for Dmin (*P* = 0.244), 0.02% ± 3.94 for Dmax (*P* = 0.959), and 0.34% ± 0.85 for Dmean (*P* = 0.721).

**FIGURE 5:**
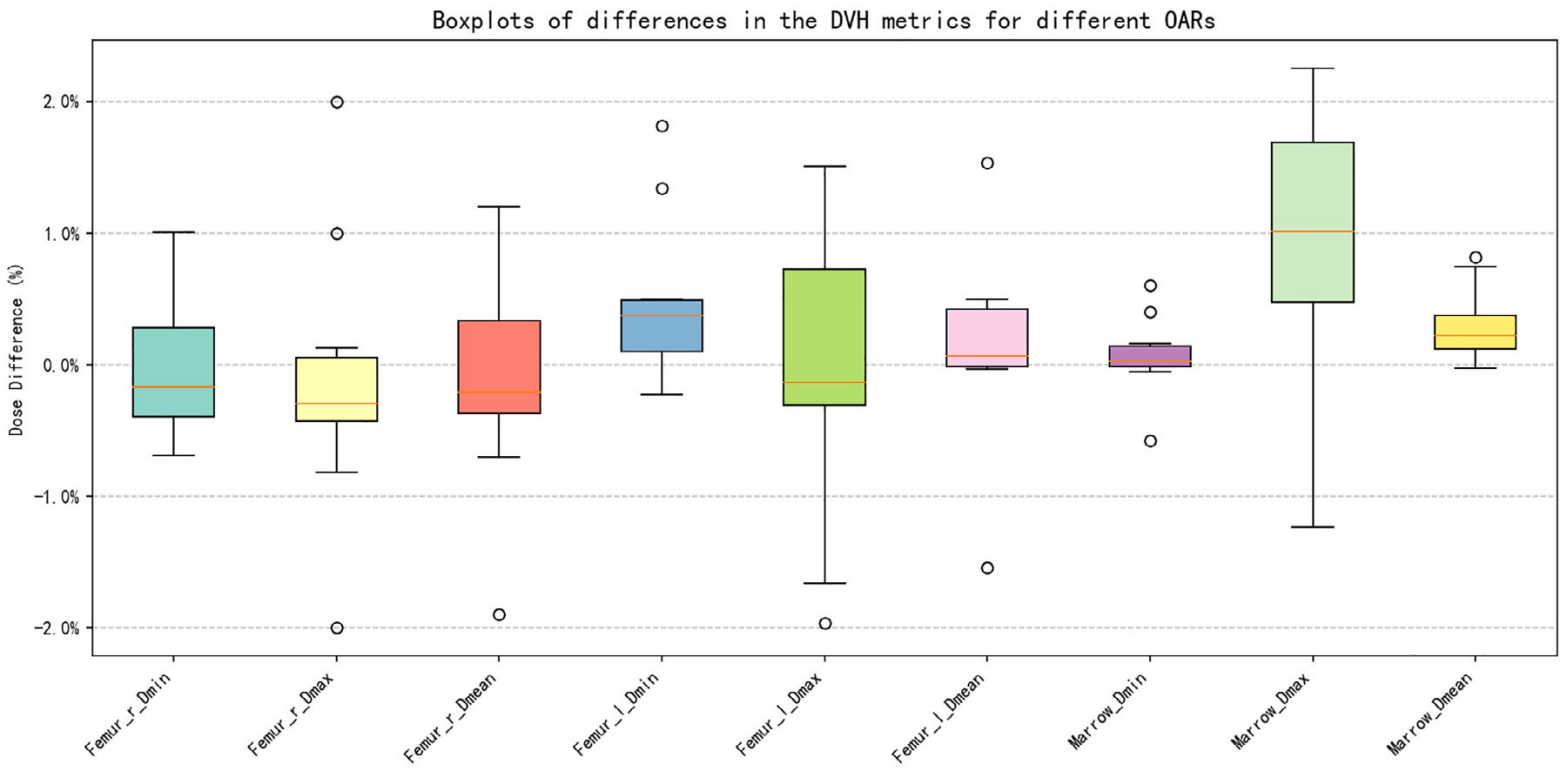
Boxplots of differences in the DVH metrics for different OARs between the pCT and sCT.

**FIGURE 6:**
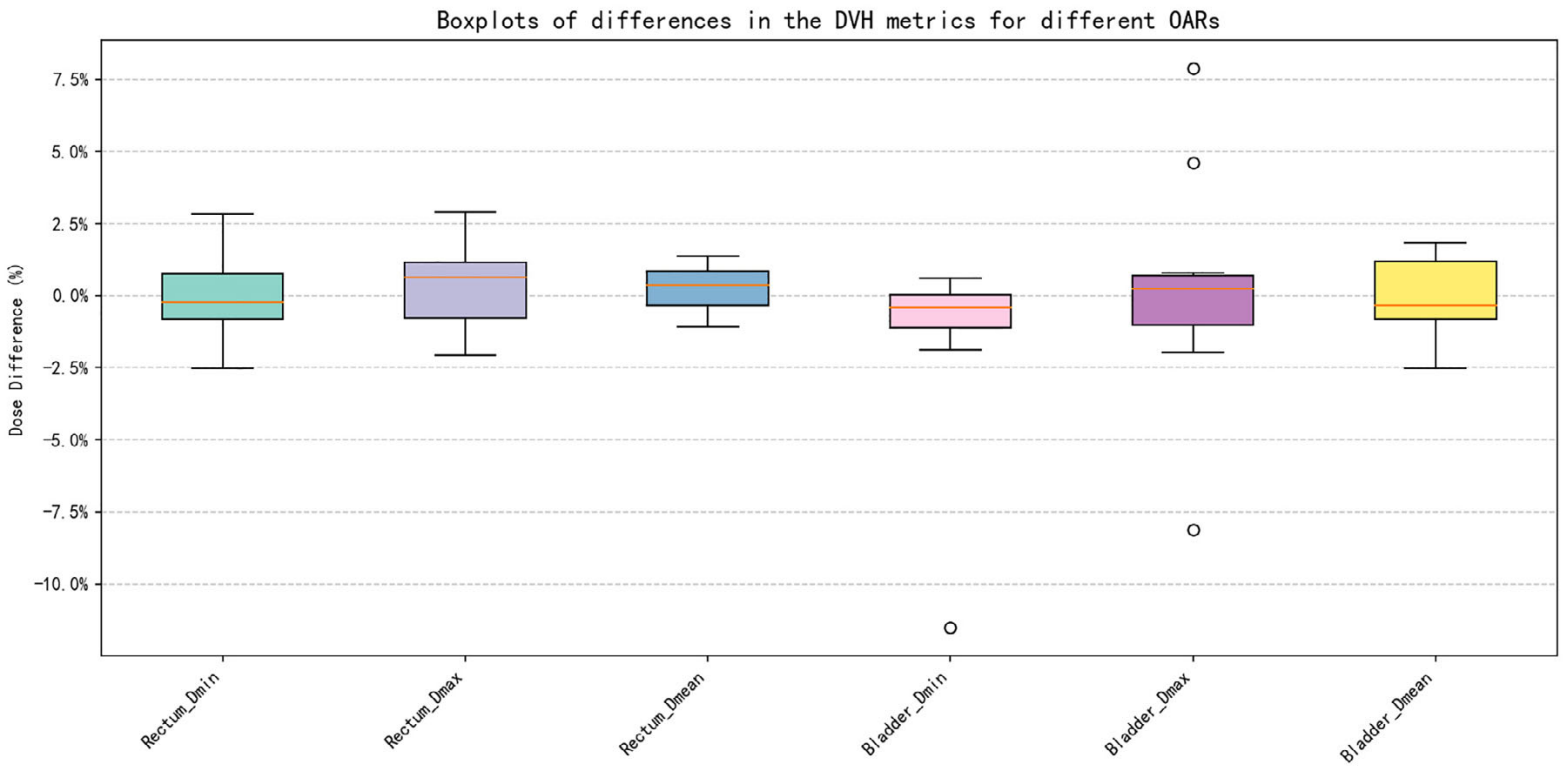
Boxplots of differences in the DVH metrics for different OARs between the pCT and sCT.

Alternatively, regarding bony OARs, for the right femoral head, the mean differences were 0.01% ± 0.54 for Dmin (*P* = 0.799), 0.02% ± 1.24 for Dmax (*P* = 0.333), and 0.06% ± 0.84 for Dmean (*P* = 0.721). For the left femoral head, the mean differences were 0.49% ± 0.74 for Dmin (*P* = 0.037), −0.13% ± 1.18 for Dmax (*P* = 0.878), and 0.05% ± 0.93 for Dmean (*P* = 0.203). For bone marrow, the mean differences were 0.06% ± 0.33 for Dmin (*P* = 0.285), 0.92% ± 1.08 for Dmax (*P* = 0.037), and 0.31% ± 0.30 for Dmean (*P* = 0.007). Although the P values for the left femoral head Dmin, bone marrow Dmax, and bone marrow Dmean were less than 0.05, indicating statistical significance, the absolute dose differences were minimal and far below the prescription dose, suggesting negligible clinical impact. No statistically significant differences were observed for the remaining OAR DVH metrics between the pCT and sCT.

### 3.5 3D γ‐analysis

The 3D γ passing rates between plans calculated on the pCT and those on the sCT are shown in Figure 7. The mean γ passing rates were 88.44% ± 2.36 under the 1%/1 mm criterion, 93.14% ± 1.93 under the 1%/2 mm criterion, 93.76% ± 1.65 under the 2%/1 mm criterion, and 95.92% ± 1.52 under the 2%/2 mm criterion.

**FIGURE 7:**
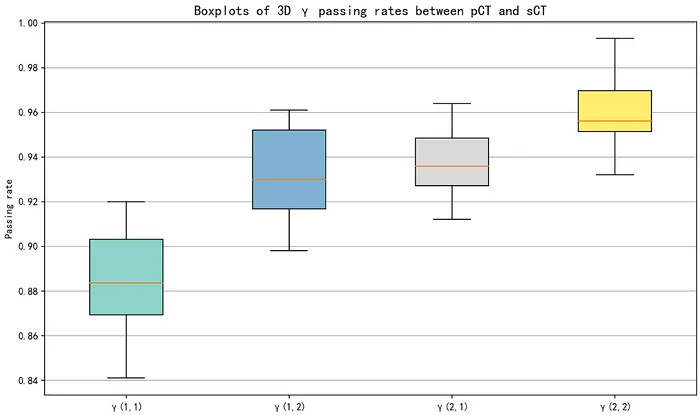
Boxplots of 3D γ passing rates between pCT and sCT.

## 4 DISCUSSION

In this study, we compared the performance of sCT images converted from MRI using a commercial deep learning model integrated in the syngo.via VB60A software platform with that of conventional CT images. Assessment was conducted in terms of HU accuracy, geometric fidelity, dosimetric parameters, and 3D γ‐analysis.

Visual inspection from Figure 1 suggests that while anatomical details on the sCT are highly comparable to those on the pCT, small differences are observed between the two datasets, especially in the bone and rectum. The subtle difference in bony structures likely stems from the fact that cortical bone produces almost no signal on MRI, and therefore the reconstruction of bony structures on the sCT is realized via atlas segmentation rather than direct mapping.^26^ Meanwhile, differences in the rectum between pCT and sCT likely originate from small residual error in image registration, which is attributed to variations in rectal filling and bowel gas between CT and MRI acquisitions. Additionally, minor blurring on the edge of bony structures is noted on the sCT. This is attributed to the adversarial loose of the cGAN, which tends to generate visually “natural” images with smooth high‐frequency details, whereas high‐contrast structures such as the cortical bone tend to display blurred edges.^34^

Similarly, comparison of the HU values of different structures between the pCT and sCT indicated that the HU values of sCT were comparable to those of pCT in soft‐tissue structures, such as the bladder and rectum. In contrast, the mean MAEs for bony structures, namely right and left femoral heads, as well as the bone marrow, were noticeably higher and exhibited greater standard deviations. This finding indicates that the deep learning model may not be as accurate in predicting HU values in bony structures compared to soft‐tissue areas. Similar observations were made in literature, where the MAE in HU were larger in bone than in soft‐tissue structures such as the bladder and rectum.^9^

Despite a larger standard deviation and greater inconsistency in the HU of bony structures, assessments of geometric fidelity suggest that for all bony structures, the DSC is consistently higher than 0.8, indicating great consistency between the pCT and sCT.^33^ This finding highlights the geometric fidelity of sCT and suggests that it is feasible to use the sCT to delineate bony structures for planning purposes. Although MRI images generally suffer from geometric distortion, a 3D distortion correction algorithm was incorporated in the MRI sequence used in the present study, thereby minimizing its impact. Additionally, efforts were made to ensure that patient positioning and immobilization were consistent between CT and MRI simulations, which further mitigated potential errors from image registration. Notably, no comparative analysis was performed for the bladder and rectum contoured on the sCT and those on the pCT. This is because CT and MRI examinations were not acquired simultaneously, and no additional patient preparation or manual intervention was implemented to standardize bowel and bladder filling. Consequently, whilst efforts were made to minimize the interval between CT and MRI acquisitions, differences in intestinal gas distribution and bladder filling between the two imaging sessions inevitably introduced anatomical inconsistencies.

Regarding dosimetric comparisons, across different OARs, variations in the DVH metrics of the bladder were the most significant. This is likely because of bladder volume variations between the pCT and sCT, highlighted by large standard deviations in the Dmin and Dmax of the bladder. Variations in bladder filling can alter both the shape of the bladder and its spatial overlap with the target volume, thereby substantially affecting DVH metrics, particularly point‐dose metrics such as Dmin and Dmax. Previous studies have similarly reported that bladder volume variability poses significant challenges for accurate prediction and dose consistency in MRI‐only workflows.^11^, ^19^, ^31^ Similarly, DVH metrics of the rectum show relatively small differences in Dmean between the pCT and sCT but noticeable fluctuations in Dmin and Dmax. These findings are likely attributable to variations in rectal gas and bowel filling, which can significantly influence local electron density distributions and consequently affect dose calculations.

Compared with soft‐tissue structures, variations in the DVH metrics of bony structures were significantly smaller, and all metrics showed a standard deviation within 1.0%. In particular, differences in the Dmean of all three bony structures were within 0.5%. This indicates that whilst bony structures exhibited higher HU variability between the pCT and the sCT, reaching up to 56 HU, the resultant dosimetric impact is negligible. This is likely attributable to the inherently high HU of bony structures, as shown in Table 1, and therefore the impact of HU variation on dose calculation is diminished.

Regarding the dosimetric parameters of PTV, differences in all of its DVH metrics remained below 1.5%, with extreme values not exceeding 3%, which is generally considered clinically acceptable in radiotherapy planning.^35^, ^36^, ^37^ Among various indicators, point‐dose metrics, namely Dmin and Dmax, showed relatively larger standard deviations, indicating that their prediction uncertainty was greater than that of volume‐based dose metrics. This finding is consistent with the intrinsic sensitivity of point doses to small geometric or density variations. While mean dose is considered more clinically relevant in assessing treatment‐related toxicity and predicting outcome, for some serial organs, accuracy in the assessment of point‐dose metrics is equally important. For such organs, the interpretation of Dmax and Dmin on the sCT requires additional caution.

3D γ‐analysis further demonstrated strong agreement between plans calculated on the pCT and those calculated on the sCT. Under the 2%2 mm γ‐analysis criteria, the mean γ passing rate reached 95.92%. Out of the 15 cases selected in this study, only 2 cases had a γ passing rate below 95% under 2%/2 mm. Further analysis indicated that the low gamma pass rate was primarily attributed to localized dose calculation discrepancies caused by inconsistent intestinal gas distributions between the CT and MRI acquisitions, as shown in Figure 8. Overall, the γ‐analysis results confirmed that the calculation accuracy of the sCT is comparable to that of the pCT.

**FIGURE 8:**
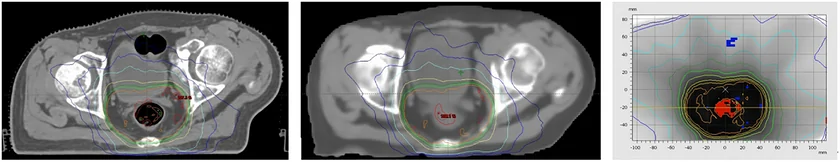
Dose distributions and γ‐analysis result of a case with a γ passing rate below 95% under 2%/2 mm. Left: dose distribution on the pCT; Middle: dose distribution on the sCT; Right: γ‐analysis map (2%/2 mm).

Collectively, the findings of this study support the feasibility of using sCT for radiotherapy planning. The MRI‐only workflow on this basis has the potential to replace the conventional CT‐based simulation workflow in selected clinical scenarios. Clinically, one of the most important advantages of the MRI‐only workflow is the elimination of systematic errors introduced by CT‐MRI image registration. Previous studies have reported that registration uncertainties may range from approximately 2 to 5 mm depending on anatomical site.^14^, ^15^, ^16^, ^17^, ^18^ Such systematic errors may persist throughout the entire treatment course, potentially leading to geometric inaccuracies and compromised tumor control probability. In addition, the MRI‐only workflow helps streamline the clinical process and reduce radiation exposure associated with planning CT scans. This is particularly relevant for pediatric patients, who are more sensitive to radiation exposure, and for patients undergoing adaptive radiotherapy, where repeated imaging is often required.

Multiple previous studies have demonstrated that the dosimetric accuracy of sCT has reached clinically acceptable levels.^9^, ^11^, ^20^, ^34^, ^38^, ^39^, ^40^ Nonetheless, several challenges remain. First, in patients with metallic implants or severe bone destruction, the HU accuracy of sCT may be compromised, which in turn affects the calculation accuracy. Second, the prediction accuracy of deep‐learning models is inherently limited by the quality and size of the training dataset. Third, quality assurance of plans generated on the sCT currently still relies on comparison with reference CT images. In a true MRI‐only clinical workflow, however, such reference CT images do not exist. Consequently, there remains a lack of patient‐specific quality assurance methods for sCT.^41^, ^42^

While this study has validated the feasibility of using sCT images converted from MRI using a commercial deep learning model integrated in the syngo.via VB60A software platform in pelvic radiotherapy planning, it has some limitations. First, the findings of the study are limited to the specific deep learning model that was assessed and cannot be generalized to other algorithms for sCT conversion. Second, as CT and MRI examinations were not acquired simultaneously, anatomical variations in certain OARs, such as the bladder and rectum, could not be eliminated, which may have consequently introduced uncertainties in subsequent dosimetric evaluations. Third, structures and plans were propagated from the pCT to the sCT using a rigid registration, which may not fully account for soft tissue deformation in the pelvic region. In future studies, we plan to expand the study cohort and apply deformable image registration to contour and plan propagation, so as to comprehensively evaluate the performance of the deep learning model in generating sCT images in the pelvic area.

## 5 CONCLUSION AND FUTURE OUTLOOK

In this study, we investigated the feasibility of an MRI‐only workflow by comprehensively evaluating the performance of sCT images converted from MRI using a commercial deep learning model integrated in the syngo.via VB60A software platform. The findings of this study suggest that the sCT generated using the deep learning algorithm demonstrates comparable accuracy to conventional CT images in terms of HU accuracy, geometric fidelity, and dose calculations. While certain limitations remain in the clinical implementation of an MRI‐only workflow based on the proposed deep‐learning sCT generation model, it has demonstrated benefits in certain clinical scenarios and warrants further research.

## AUTHOR CONTRIBUTIONS

Feng Lin conceived and designed the project. Feng Lin and Jinyan Hu acquired the data. Zhao Li and Jun Wang performed target and organ‐at‐risk delineations required by the study. Wenying Zhang provided relevant clinical cases. Feng Lin, Aiping Wen and Mao Li analyzed and interpreted the data. Feng Lin wrote the manuscript with input from all authors. Yunfei Hu reviewed the manuscript. Yang Wang supervised the project and reviewed the manuscript.

## CONFLICT OF INTEREST STATEMENT

Yunfei Hu has received travel costs and honoraria for presenting on behalf of Varian Medical Systems, which is part of Siemens Healthineers.

## ETHICS STATEMENT

Ethical approval is not applicable for this article.

## Data Availability

The data that support the findings of this study are available from the corresponding author upon reasonable request.
